# Oro-facial malignancy in north of Iraq: a retrospective study of biopsied cases

**DOI:** 10.1186/s12903-021-01521-3

**Published:** 2021-03-23

**Authors:** Sura Ali Ahmed Fuoad, Dena Nadhim Mohammad, Marwa Abdul-Salam Hamied, Balkees Taha Garib

**Affiliations:** 1 Oral Medicine, Diagnostic and Surgical Dental Science Department, College of Dentistry, Gulf Medical University, Ajman, UAE; 2 Oral and Maxillofacial Pathology, Oral Diagnosis Department, College of Dentistry, University of Sulaimani, Sulaimani, Kurdistan Region Iraq

**Keywords:** Oral cancer, Squamous cell carcinoma, Tongue, ICD-10, Sualimani

## Abstract

**Background:**

Identifying the prevalence of orofacial malignancies is essential to provide health-care programs and services for a specific population. This study aimed to specify the prevalence, clinical and histopathological features of oral malignancies in Sulaimani for over 12 years.

**Methods:**

All archived reports for orofacial specimens from 2008 to 2019 were collected from three major centers in Sulaimani governorate. The demographic data, date, type of surgery, lesion's site, and diagnosis were recorded. The ICD-10 coding was specified for each case. A Chi-square test was used to assess differences between genders. A one-way ANOVA test was applied to analyze the differences in mean age distributions among different sites of oral malignancies and oral squamous cell carcinoma histopathological grades. *P* < 0.05 was considered significant.

**Results:**

Orofacial malignant lesions account for 14.53% of the total sample. Patients' mean age was (55.46 ± 18.48) years, and they were predominantly males (M: F ratio = 1.15:1). The tongue was the frequently affected site (14.8%). A Chi-square test showed no significant difference between genders concerning age (*P* = 0.118), years of registration (*P* = 0.28), and site (*P* = 0.29). The C06 (represents; cheek mucosa C06.0, the vestibule of mouth C06.1, retromolar area C06.2, and other unspecified parts of mouth C06.8) was the most frequent topographical ICD-code (18.1%). Carcinoma was a frequent malignancy (63.2%). OSCC was the most common lesion (56.4%). It commonly affects males, and the tongue was the frequent site (24.1%) followed by lip (17.8%).

**Conclusions:**

There is a slight increase in the registered oral malignancies in Sulaimani city over 12 years; they were predominated in males, in 61–70 years old patients, and being oral squamous cell carcinoma types.

## Background

The oral mucosa can be affected by non-neoplastic and neoplastic lesions. It reflects or manifests the underlying systemic disease as oral health is integral with general health [1]. Oral cancer is increasing in the annual incidence of > 300,000 cases, with a high mortality rate reported in less developing countries. It is attributed to the accumulative factors throughout population aging and cancer-provoking factors expansion [2].

Malignant lesions in the orofacial region vary in their causative factors, genetic predisposition, origin, location, and histopathological differentiation [3]. Furthermore, the demographic variation (age and gender) and site preference are crucial to determine their characteristics [4]. Annual estimation of orofacial malignancy prevalence is essential to identify the risk population and emphasize health-care programs and services [4].

Taking biopsy is essential to establish a definitive diagnosis based on the lesion histological characteristics and in correspondence to their clinical presentation. It gives baseline information about the disease distinction, prognosis, and prevalence. Thereby it facilitates planning a proper management strategy. Biopsy acknowledges the categorization of lesions in a coherent and higher systematic way. Besides, the biopsy is considered undeniable medico-legal merit [5].

A limited number of studies were conducted in different Middle East countries regarding histopathologically confirmed orofacial lesions, and few studies estimated oral malignancies relative frequency [6–14]. Oral squamous cell carcinoma (OSCC) is the commonest oral malignancy. It is prevalent in middle-aged and elderly, although it has been reported in young adults [15]. Analysis of data from the Iraqi Cancer Registry includes all cases reported in Iraq except the Kurdistan region (north of Iraq), revealed that oral cancer accounts for 2% of all cancers reported in 2000–2008, and 91% of cases were OSCC [16]. In 2008, a large epidemiological retrospective study was conducted on 1,425 biopsy reports in Iraq. It showed that OSCC was frequently reported in over 50 years old patients with a male predilection [17]. In another epidemiological study in Basrah city- south of Iraq, OSCC reported in 21 cases with males preference, and the tongue was the commonly affected site [18]. Histopathological record studies were done in Sulaimani, Mosul, Baghdad, and Basrah cites of Iraq, in which different oral malignancies frequencies were registered [19–23].

From a systematic review of 82 hospital records over 26 years in Saudi Arabia, data revealed a high prevalence of oral cancer, ranging from 21.6% to 68.6% [24].

The present study aimed to determine the orofacial malignancies surgical specimen distributions concerning age, gender, year of registration, lesion's site, disease ICD classification, and histopathological typing in Sulaimani governorate. Besides the estimation of gender variations in these different parameters. Then specify those parameters in OSCC alone.

## Materials and methods

The current retrospective study analyzed the clinico-pathological reported archives of surgical specimens excised for confirming the diagnosis of oro- facial lesions. The data was collected from three major centers in Sulaimani governorate, north of Iraq, Kurdistan region; College of Dentistry-Sulaimani University, and two primary referral histopathological laboratories in government hospitals (Shorsh Hospital- Ministry of Military Defense) and Shahid Saifaldeen Hospital—Ministry of Health). The Research Ethics Committee in the College of Dentistry/Faculty of Medical Sciences, Sulaimani University approved the study (Proposal No. 199, 20/9/2020). All methods were performed following the relevant guidelines and regulations. Informed consent had been waived by the Research Ethics Committee in the College of Dentistry/Faculty of Medical Sciences, Sulaimani University.

A total of 2319 histopathological archive reports of oral lesion surgical specimens recorded between 2008 and 2019 were retrieved. The inclusion criteria include all malignant lesions of the orofacial region and neck. Duplicated cases, re-excised surgical samples, unclear or incomplete data, and inconclusive diagnosis were the exclusion criteria. All data generated or analyzed during this study were included in this published article is shared as supplementary information files.

Information about age, gender, laboratory name, year of registration, type of surgical biopsy, site. The authors specified the topographic code of ICDO classification for oncology. It was identified for all cases based on the World Health Organization International Statistical Classification of Diseases for Oncology ICD-10 (WHO 2016a) and Related Health Problems 10th Revision 2010-II Neoplasms [25]. Finally histopathological diagnosis (with grading if present) was registered.

The data set was tabulated in an Excel worksheet and statistically analyzed using the Statistical Package for the Social Sciences software (SPSS Version 16.0. Chicago, SPSS Inc.). The frequency and percentage distributions were calculated for nonparametric variables (age-group, gender, years of registration, site, ICD coding system, and malignant histological groups) and analyzed by Pearson Chi-square (χ^2^) test for gender's differences. The mean and standard deviation of the age was calculated. A one-way ANOVA and Post Hoc Tukey test was used to analyze the variances in mean age among different sites and OSCC grades. A *P* value of 0.05 was used as a cut-off point for the test significance.

## Results

**Fig. 1:**
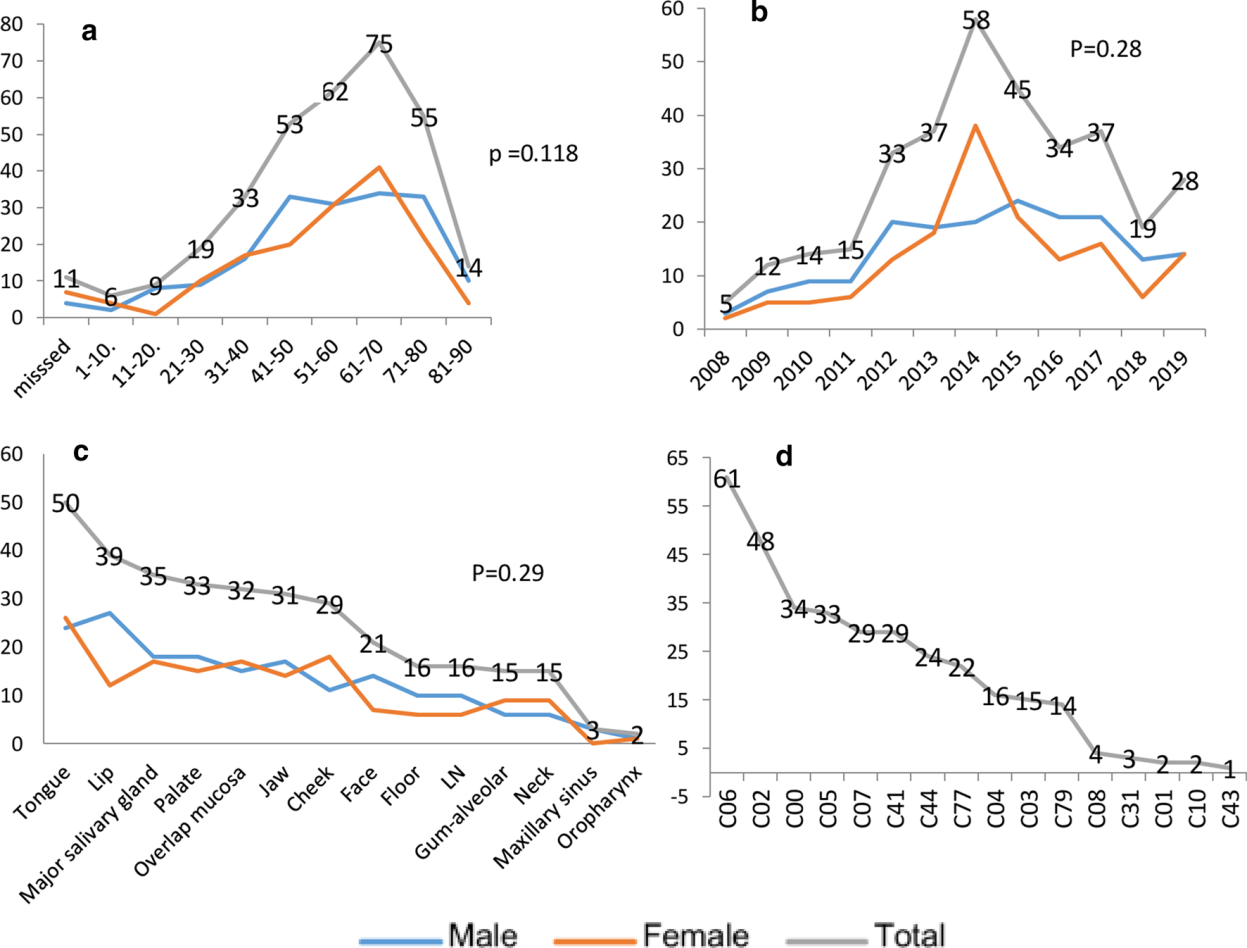
Frequency distribution of oral-facial malignant neoplasm in Sulaimani city according to the age group (**a**), years of registration (**b**), anatomical site (**c**), and ICD-10 coding for the total sample (**d**). Pearson Chi-Square test (*P* > 0.05) between genders

Out of 2319 histopathological archive reports of orofacial specimens recorded, there were 337 (14.5%) reports with definitive orofacial malignancy over the twelve years (2008–2019) in Sulaimani city. The age ranged from 1–90 years old, with a mean (55.46 ± 18.48) years. The majority of cases (22.3%, n = 75) located in the (61–70) age group, followed by 18.4% (n = 62) in the age group (51–60). (Fig. 1a). Malignancy occurred in males 53.4% (n = 180) more than in females 46.6% (n = 157).

Sixty-one samples (18.1%) were incisional biopsy; the remaining included excisional surgical samples. The peak number of recorded orofacial malignancies was in 2014 (17.2%, n = 58), followed by 2015 (13.4%, n = 45). The least were in 2008 (1.5%, n = 5) (Fig. 1b). Furthermore, younger patients aged less than 20 years were more frequent in males (10 cases versus 5). Detailed analysis showed that patients within 41–60 years had frequent malignant neoplasms of the tongue.

The tongue was the most frequently affected site 14.8% (n = 50), then lip 11.6% (n = 39), and 10.4% (n = 35) of cases in major salivary glands. Females showed slightly more frequent malignancies in the cheek (M: F ratio = 1:1.6), and male predominance was seen in the lip, face, and floor of mouth with male to female ratio (2.25:1), (2:1), and (1.66:1), respectively (Fig. 1c). The Chi-square test showed no significant difference between genders concerning age-groups (*P* = 0.118), the years of registration (*P* = 0.28), and anatomical site (*P* = 0.29) (Fig. 1).

Concerning the topographical code of malignant lesions showed that (C06) (represented; cheek mucosa C06.0, the vestibule of mouth C06.1, retromolar area C06.2, and other and unspecified parts of mouth C06.8) was the most frequently involved site 18.1% (n = 61). Tongue malignancy (C02) was the next frequently detected 14.2% (n = 48). It included; C02.0 dorsal surface, C02.1 border of the tongue, C02.2 ventral surface, C02.8 overlapping sites, and C02.9 unspecified site of the tongue. The predominant sub-site was the border of the tongue (7.7%, n = 26). The lip accounted (10.1%, n = 34), followed by the palate 9.8% (n = 33). Secondary malignancy accounted for 36 cases (10.7%), mainly in lymph nodes (C77.0) 22 cases. C43 (malignant melanoma of skin) was the least 0.3% (n = 1). (Fig. 1d, Table 1).

**Table 1:** Topography distribution according to the ICD-10-CM Diagnosis Code in the definition of oral malignant neoplasms for 337 cases

ICD	Topographic location	No	%	ICD		No	%
C00	Lip	34	10.1	C00.0	External upper lip	1	.3
				C00.1	External lower lip	22	6.5
				C00.3	Upper lip, inner aspect	1	.3
				C00.4	Lower lip, inner aspect	5	1.5
				C00.5	Unspecified, inner aspect	1	.3
				C00.6	Commissure of lip	4	1.2
C01	Base of tongue	2	.6	C01	Base of tongue	2	.6
C02	Other and unspecified parts of the tongue	48	14.2	C02.0	Dorsal surface of tongue	5	1.5
				C02.1	Border of tongue	26	7.7
				C02.2	Ventral surface of tongue	1	.3
				C02.8	Overlapping sites of tongue	4	1.2
				C02.9	Tongue, unspecified	12	3.6
C03	Gum and alveolar mucosa	15	4.5	C03.0	Upper gum	4	1.2
				C03.1	Lower gum	5	1.5
				C03.9	Gum, unspecified	6	1.8
C04	Floor of mouth	16	4.7	C04.0	Anterior floor of mouth	1	.3
				C04.9	Floor of mouth, unspecified	15	4.5
C05	Palate	33	9.8	C05.0	Hard palate	10	3.0
				C05.1	Soft palate	6	1.8
				C05.9	Palate, unspecified	17	5
C06	Other and unspecified parts of the mouth	61	18.1	C06.0	Cheek mucosa	20	5.9
				C06.2	Retromolar area	6	1.8
				C06.8	Overlapping sites of other and unspecified parts	31	9.2
				C06.9	Mouth, unspecified	4	1.2
C07	Parotid gland	29	8.6	C07	Parotid gland	29	8.6
C08	Other and unspecified major salivary glands	4	1.2	C08.0	Submandibular gland	2	.6
				C08.1	Sublingual gland	1	.3
				C08.9	Major salivary gland, unspecified	1	.3
C10	Oropharynx	2	0.6	C10.2	Lateral wall of oropharynx	2	.6
C31	Maxillary sinuses	3	.9	C31.0	Maxillary sinus	3	.9
C41	Bone	29	8.6	C41.0	Bones of skull and face	16	4.7
				C41.1	Mandible	13	3.9
C43	Malignant melanoma of skin	1	.3	C43.9	Malignant melanoma of skin, unspecified	1	.3
C44	Other malignant neoplasms of the skin	24	7.1	C44	Other and unspecified skin	1	.3
				C44.01	Skin of lip	5	1.5
				C44.31	BCC skin of other and unspecified parts of face	9	2.7
				C44.320	SCC of skin of unspecified parts of face	8	2.4
				C44.399	Other specified of skin of other parts of face	1	.3
C77	Secondary	22	6.5	C77.0	Unspecified of lymph nodes	22	6.5
C79		14	4.2	C79.2	Skin	2	.6
				C79.51	Bone	1	.3
				C79.89	Other specified sites	11	3.3

**Fig. 2:**
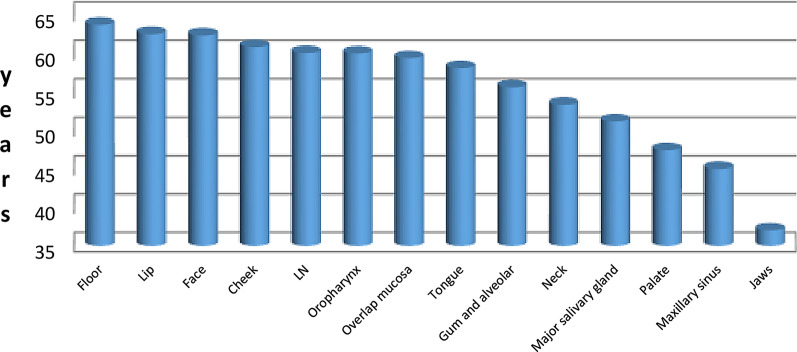
The mean age distribution in relation to the site of orofacial malignancy. *Mean age was significantly more for jaw lesions (One-way ANOVA test and post hoc test* P* = 0.000)

All anatomical sites of malignant lesions showed an almost similar mean of age except those at the jaws. They occurred at a significantly younger age (one- way ANOVA and post Hoc test *P* = 0.000) (Fig. 2).

**Fig. 3:**
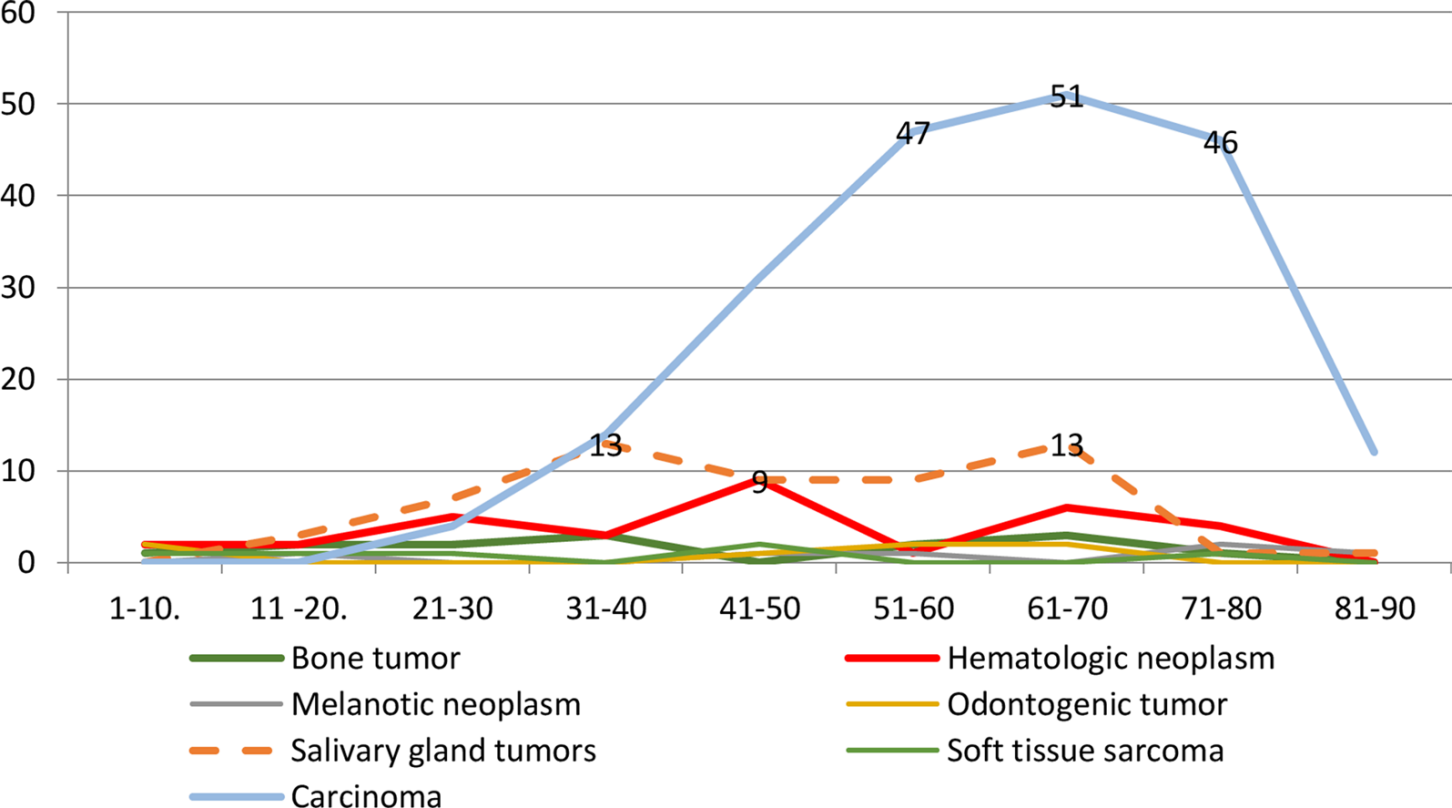
Frequency distribution of different oral-facial histological malignant groups according to the age group. Pearson Chi-Square test (*P* = 0.000) difference in frequency among age-groups

Regarding the malignancy histogenesis, carcinoma was the most frequent malignancy (63.2%), followed by salivary gland tumors (17.5%). Soft tissue sarcomas and melanotic neoplasm were the lowest orofacial malignancy, 1.8%. Accordingly, OSCC was the most common lesion 56.4% (n = 190), followed by adenoid cystic carcinoma 7.1% (n = 24) (Table 2). Orofacial malignancies had a highly significant difference in their distribution with patients' age-groups (Chi-Square test, *P* = 0.000). Thus, carcinomas had a peak of (61–70) years age-group (n = 51). Salivary gland tumors had two peaks (each included 13 patients); an earlier one at (31–40) years group and a later one at group (61–70) years. The hematogenic neoplasms' peak was seen in the age-group (41–50) years (n = 9) (Fig. 3).

**Table 2:** Frequency and percentage distributions of histopathological diagnosis of malignant lesions

	Diagnosis	No	%
Hematologic neoplasm (32, 9.5%)	Lymphoma	17	5.04
	Non-Hodgkin’s lymphoma	6	1.78
	Diffuse large B-cell lymphoma	2	0.59
	Burkitt’s lymphoma	1	0.3
	Multiple myeloma	1	0.3
	Langerhans cell histiocytosis X	4	1.19
	Plasma cell tumor	1	0.3
Bone tumor (14, 4.2%)	Spindle cell sarcoma	1	0.3
	Ewing sarcoma	1	0.3
	Osteochondrosarcoma	2	0.59
	Osteosarcoma	10	2.97
Melanotic (6, 1.8%)	Melanoma	6	1.8
Odontogenic (7, 2.1%)	Melanotic neuroectodermal tumor	2	0.59
	Ameloblastic carcinoma	5	1.48
Soft tissue sarcoma (6, 1.8%)	Rhabdomyosarcoma	2	0.59
	Spindle cell sarcoma	1	0.3
	Malignant fibrous histiocytoma	1	0.3
	Angiosarcoma	1	0.3
	Small cell tumor	1	0.3
Salivary gland tumors (59, 17.51%)	Adenoid cystic carcinoma	24	7.12
	Mucoepidermoid carcinoma	15	4.45
	Polymorphous low-grade adenocarcinoma	8	2.37
	Acinic cell carcinoma	3	0.89
	Carcinoma ex pleomorphic adenoma	3	0.89
	Mammary analogue secretory carcinoma	2	0.59
	Epithelial-myoepithelial carcinoma	1	0.3
	Myoepithelial carcinoma	1	0.3
	Intraductal carcinoma	1	0.3
	Papillary carcinoma	1	0.3
Carcinoma (213, 63. 24%)	Squamous cell carcinoma	190	56.4
	Basal cell carcinoma	11	3.3
	Hutchinson’s freckle	1	0.3
	Verrucous carcinoma	6	1.78
	Metastatic carcinoma	1	0.3
	Nasopharyngeal carcinoma	1	0.3
	Undifferentiated carcinoma	3	0.89
Total		337	100

**Fig. 4:**
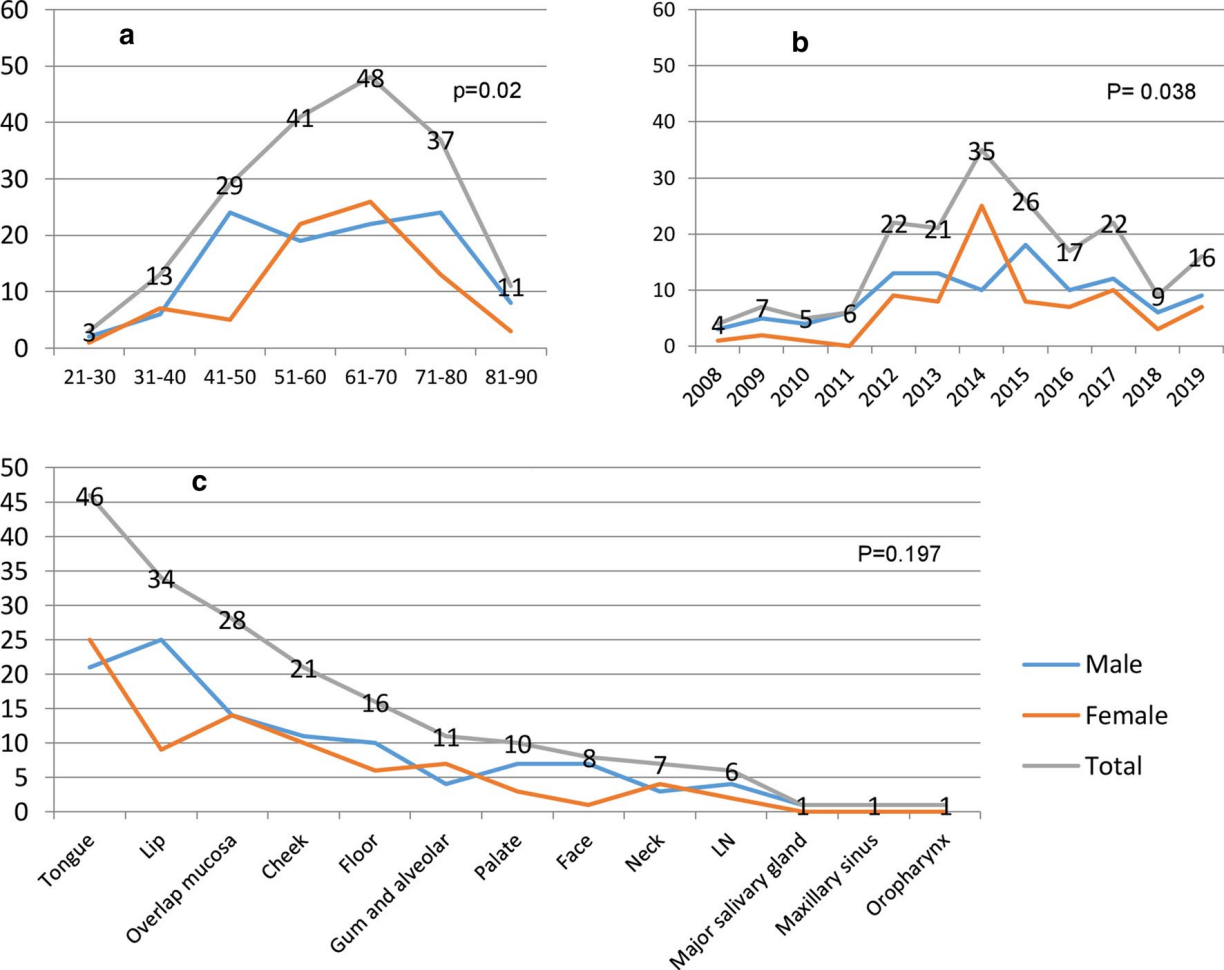
Frequency distribution of OSCC concerning age (**a**), year of registration (**b**), and location (**c**) in the total sample and both genders. Pearson Chi-Square test (*P* value) between genders

Detailed information about OSCC indicated that it was frequently found in males (109 versus 81 in females). The mean age of the patients was (61.03 ± 14.24) (Table 3), most of the cases registered in 2014, and the tongue is on the top of affected sites (46, 24.1%), followed by the lip (34,17.8%) (Fig. 4). There were significant statistical differences between genders concerning age and year of registration. Although the peak of the age of occurrence was nearly equal in both genders, females were less at (41–50) and (81–90) age-groups (chi-square test, *P* = 0.02) (Fig. 4a). Again females express a high frequency in 2014 (n = 25), while males showed a high frequency in 2015 (16.5%, n = 18) (chi-square test *P* = 0.038) (Fig. 4b). Although SCC of the lip was a common site in males (22.9%, n = 25), there were no gender differences in OSCC site distribution (chi-square test *P* = 0.197) (Fig. 4c).

**Table 3:** Frequency and percentage distribution of 190 cases of OSCC according to the histopathological grading concerning mean age and gender

Histopathological grading	No	%	Age	ANOVA*	Male		Female		X^2^- test**
			Mean ± SD		No	%	No	%	
Well	113	59.5	60.7 ± 14.7	*P* = 0.92	60	53.1	53	46.9	*P* = 0.45
Moderate	57	30	61.2 ± 14.05		36	63.2	21	36.8	
Poor	19	9.9	62.4 ± 12.28		12	63.2	7	36.8	
Undifferentiated	1	0.5	54		1	100	0	0	
Total	190	100	61.03 ± 14.24		109	57.4	81	42.6	

^*^One-way ANOVA test, among the mean age values of different histopathological grades groups

^**^Pearson Chi square test, variation in gender distribution among different histopathological grades groups

Nearly half of OSCC cases were well-differentiated (59.5%, n = 113), only 9.9% were poorly-differentiated. The statistical analysis showed no significant relationship between histopathological grading of OSCC between genders (Chi-square test *P* = 0.45) or mean age (*P* = 0.92, one-way ANOVA test) (Table 3).

## Discussion

A wide range of lesions can affect the orofacial region. They may have a similar clinical presentation, even though some of them being malignant and life-threatening. In many cases, a biopsy is essential to establish an accurate diagnosis and subsequent proper management. Also, retrieving the reports of surgical biopsies for malignant neoplasm can direct vision to assess this health problem. The majority of our cases underwent excisional removal to establish a definitive diagnosis. However, different pathologists used different words to describe similar lesions. Reevaluation of histopathological description was essential to unify the diagnostic terms.

Our result indicated a slight increase in the rate of oral malignancies over time (14.5%) compared to a five-year (2004–2009) retrospective study (12.3%) in Sulaimani [19] and another previous study in Baghdad during 1991–2000 (9.97%) [26]. This could be attributed to pollution and recurrent explosions, and wars in Iraq. Our result was similar to that reported in UAE (14.9%) [8], but lesser than that reported in Basrah-south of Iraq (19.1%) [23], and higher than in KSA (5.7%) [13], Iran (2.39%) [27], (3.88%) [28].

Oral malignancy occurred at a wide age range. In this study, it ranged from 1–90 years old. The highest age-group (61–70) was similar to results from Sulaimani [19] and Basrah cities [23]. However, the patients were older than those reported in two other Iraqi studies of OSCC (51–60) [29], (50–69) [30].

In this study, the mean age of oral malignancy (55.46 ± 18.48) was similar to the mean age of SCC patients in UAE (54.9 ± 12.9) [8] but higher than in KSA (49.5 ± 20.7) [13]. The present study showed that carcinomas were seen in the fifth to seventh decades. In comparison, a Saudi Arabian study reported higher mean age of oral malignancy (64.8) [11]. This variation could be due to variable reactions in older people [10, 18, 24] prolonged and accumulative, intense exposure to various provoking factors.

This study also showed that specific demographical findings might characterize different age-groups. Younger patients with age less than 20 years were twice as frequent males, which agrees with Al- Reyahi's study [26]. However, our cases aged within (41–60) years showed tongue malignancy and contradicted the above Iraqi study as teenage patients suffered predominantly from tongue and lip cancers [26].

Biopsy records revealed that the male gender predominated [26, 28]. The current study also showed male predominance (M: F ratio = 1.15:1), which disagreed with the finding in Sulaimani in Khudier's study concerning oral malignancy (M: F ratio = 0.9:1) [19]. Studies from different Iraqi governorates declared a higher male ratio (M: F ratio = 1.37:1) [16], (1.38:1) [23], OSCC study (1.71:1) [29], (1.4: 1) [30]. In KSA, the M: F ratio in Jeddah varied from (0.8:1) [24] to (1.26:1) [13]. In Sudan, the ratio was 3:2 [10]. Such findings might be attributed to the expanded presentation to risk factors by the males [23] like smoking, alcohol, sun exposure, or even hormonal factors. Further analysis of the gender variation of the affected site showed differences. Cheek was the predominant site in females, while lip, face, and mouth floor were predominant in males. On the contrary, Museedi and Younis found men significantly more affected with lip cancer, tongue, gum, mouth palate, and other sites of mouth than women [16]. However, lip and palatal malignancies were more in females, and tongue cancer was more in males [26].

The current study result aligned with the most Iraqi studies stated that the tongue was the mainly affected site (42.7%) [16], (46.7%) [23]. In Sudan, the location of overlap areas of the oral cavity was on the top (38.7%), then tongue (9.2%), lip (5.4%) [10]. Moreover, Dhanuthai et al. reported that the tongue was the most frequently involved site (25.4%) then labial/buccal mucosa (21.7%) [28]. The recorded clinical features of different studies that depend on biopsied oral-facial malignancies in different countries were shown in Table 4.

**Table 4:** The clinical features of biopsied oral-facial malignancies in different countries in the Middle East

	Country	Author(s)	Year	M:F	Prevalence	Age^$^	Most affected oral site
Oral malignancies	Iraq	Al–Niaimi [20]	2006	1.2:1	2%	58.3	Lip 41.1%, tongue 23.5%
		Khudier [19]	2012	0.9:1	12.3%	–	–
		Hassawi et al. [21]	2010	–	12.8%*	–	–
		Mohammed [22]	2014	1.56:1	0.12%	39.1	Perioral 39.5%, buccal mucosa 18.6%
		Museedi and Younis [16]	2014	1.37:1	2%	> 60	Tongue > lip
		Aljazaeri et al. [23]	2020	1.38:1	19.1%	> 69	Tongue 46.7%
	UAE	Anis and Gaballah [8]	2013	–	14.9%	–	–
	Yemen	Halboub et al. [9]	2011	1:1	4%	58.4	Tongue 29.9%, floor 10.2%
	Sudan	Osman et al. [10]	2010	3:2	50%	> 50	Overlap 38.7%, tongue 9.2%
	Saudi Arabia	Ali et al. [12]	2013	1.42:1	9.9%	45.4	
		Saleh et al. [11]	2017	0.6:1	38.8%	64.8	Tongue 43.7%, buccal mucosa 26.4%
		AlHindi et al. [13]	2019	1.26:1	5.7%	49.52	–
	Kuwait	Ali and Sundaram [6]	2012	–	55.6%	–	–
		Joseph et al. [7]	2019	1.4:1	3.6%	51.29	–
	Jordan	Telfah and Hammouri [14]	2014	1.6:1	1.5%	–	–
OSCC	Iraq	Al–Niaimi [20]	2006	1.2:1	80%	–	Lip 51.5%,tongue 29.4%
		Khudier [19]	2012	1.2:1	56.1%	50–70	Lip 50%, tongue20%
		Hassawi et al. [21]	2010	3:1	58.9%	–	–
		Mohammed [22]	2014	–	49%	–	–
		Museedi and Younis [16]	2014	–	91%	–	–
		Aljazaeri et al. [23]	2020	1.36:1	84.1%	–	–
	UAE	Anis and Gaballah [8]	2013	4.13:1	77%	54.9	Tongue 51.9%, buccal mucosa 19.48%
	Yemen	Halboub et al. [9]	2011	–	93.2%	–	–
	Sudan	Osman et al. [10]	2010	–	73.6%	> 50	–
	Saudi Arabia	Ali et al. [12]	2013	1.37:1	18.2%	60.39	–
		Saleh et al. [11]	2017	0.54:1	93.1%	65.1	Tongue 43.7%, buccal mucosa 26.4%
		AlHindi et al. [13]	2019	1.4:1	65.7%	56.6	–
	Kuwait	Ali and Sundaram [6]	2012	1:1	44.4%	53.9	Buccal mucosa 39%, tongue28.5%
		Joseph et al. [7]	2019	–	64%	–	–
	Jordan	Telfah and Hammouri [14]	2014	–	67.3%	–	Lip 23.7%, tongue 18.6%

^$^Mean of age, – not mentioned

The International Classification of neoplasms had been used in this study with its subdivisions to convey a standardized, consistent universally, systematic language for reporting site of malignancy and comparison of data lifelong. There is no published literature based on ICD-10 categories that analyzed the malignant neoplasms on a large scale in the north of Iraq. This study indicated that the C06 (represents; cheek mucosa C06.0, the vestibule of mouth C06.1, retromolar area C06.2, and other unspecified parts of mouth C06.8) was the most frequently coded site (61, 18.1%) and tongue malignancy (2nd most affected site) since the later was divided into a base of the tongue (C01, 0.6%) and others and unspecified parts of the tongue ( C02, 14.2%) (Dorsal surface C02, border C02.1, ventral surface C02.2, Overlapping sites C02.8, unspecified C02.9). Most of the previous local and global studies depend on the neoplasm's anatomical localization and did not follow the ICD coding. Except for Al- Reyahi [26], who found that the lip (C00) was the most frequent site, while Museedi and Younis [16] in their study reported that the tongue and other (ICD-02) is the predominant site.

Regarding OSCC, in UAE (51.9%) of cases located in tongue followed by cheeks (19.48%), lips (11.6%) [8]. A previous study in Sulaimani found (50%) of cases affected the lip and (20%) detected in the tongue [19], whereas in our study, SCC of the tongue is ahead (24.21%) of the lip (17.89%). Iraqi studies showed different frequencies of OSCC (91%) [16], (16.1%)from all other lesions, and 84.1% from malignant lesions [23].

Regarding malignant origin, in the present study, carcinomas had the commonest origin (63.24%), followed by glandular parenchyma (17.51%). Thus, OSCC was the most common (56.4%) malignancy, then adenoid cystic carcinoma (7.1%). Our results confirmed previous studies for the rank of the diagnosed histopathological malignancy. Khudier [19] found OSCC in (56.1%) cases, then salivary gland carcinoma (19.6%), similar to our finding.

Dhanuthai et al. showed that the majority of oral cancer arose from epithelial, which was (85.09%) and (80.05%) of them were OSCC [28]. In Sudan, OSCC accounts (73.6%) of all oral malignancies, followed by verrucous carcinoma [10]. In UAE [8] registered (77%) SCC cases from oral malignancies. Al-Hindi et al. [13] reported SCC in (65.7%). Moridani et al. found OSCC in (1.52%) of oral cancer, followed by salivary gland malignancies (0.86%) [27]. The clinicopathological distributions of OSCC in middle east countries were shown in Table 4. Another study on East African pathological records reported that OSCC was (15.22%) of all the oral maxillofacial neoplasms [31].

In the current study, most OSCC cases were well-differentiated (59.5%), followed by moderately differentiated SCC (30%). In UAE, (62.3%), (20.8%), (6.5%) accounted for well, moderate, and poorly differentiated OSCC, respectively [8]. In Sudan, the well-differentiated SCC was the most prevalent (62.3%), followed by moderated differentiated SCC (20.8%) [10]. This grading finding supports other studies in Iraq [17], UAE [8].

According to this study, there was an increase in the registered orofacial malignancies in Sulaimani city. This rise may indicate a good health service in the region that attracts the surgeons to submit their surgical samples to these centers and do not send them to the capital Baghdad. Besides, the accumulative effects of pollutions, which is considered an additive provoking factor, are expressed over time. Thus, people in the north of Iraq might have orofacial malignancies risk and need educational programs to promote preventive measures and early detection and management.

## Data Availability

*Material form* all archived data of patients reports from 2008 to 2019 with orofacial malignancies were recorded; *Location of material* The data was collected from three major centers in Sulaimani governorate, north of Iraq; College of Dentistry-Sulaimani University, and two primary referral histopathological laboratories in government hospitals (Shorsh Hospital- Ministry of Defence Affairs) and Shahid Saifaldeen Hospital—Ministry of health); *the material can be accessed* The datasets used and analyzed during the current study are available from the corresponding author on reasonable request.
